# Reducing Cost of Care Through Deimplementation of Surgical Overtreatment

**DOI:** 10.1097/AS9.0000000000000089

**Published:** 2021-09-02

**Authors:** Heidi Nelson

**Affiliations:** 1 From the Division of Research and Optimal Patient Care, Cancer Department, American College of Surgeons, Chicago, IL.

The cost of healthcare has been steadily rising as a percentage of the United States Gross Domestic Product (GDP) from a low of 5% in 1960 to 12% in 1990 and 18% of GDP as of 2019.^1^ While this has been a subject of national interest and grave concern for as long as the immutable trend has been observed, we are now starting to appreciate just how much the cost of care interferes with the health and well-being of our patients. In cancer care, for example, it is not uncommon for patients and their families to experience financial toxicity, and bankruptcy during the most vulnerable time of their cancer care journey. Unfortunately, the steady growth of healthcare costs feels to medical professionals like a formidable challenge that is outside their control. Despite this, we must involve ourselves and our professional organizations, if for no other reason than to serve the needs of our patients. While we may not be able to change the price of drugs or devices, if there is any good news in this financial story, it is that we can have a direct impact on cost of care by reducing healthcare waste. Waste in healthcare is estimated as representing 30% of expenditures and this includes $12.8 to $28.6 billion spent on overtreatment or low-value care.^2^ The possibility that surgeons can reduce healthcare expenditures by challenging assumptions about the value of routine surgical practices and reducing overtreatment is discussed in a report by Lin et al,^3^ in this issue of *Annals of Surgery*.

Lin et al^3^ reviewed the diverticulitis literature that challenged the practice of routine colectomy following successful medical management of acute disease, and led to the new guidelines recommending case-by-case decision-making. They studied inpatient expenditures before and after the guidelines were published and reported that total savings that may be associated with postguideline implementation, was $6.36 million, per quarter. So how do we get from millions to billions of dollars of savings through deimplementation of low value practices? Logically we need to identify many more low-value and overtreatment practices, and we need to understand what it takes to accelerate the translation of new knowledge into practice.

Until recently, many of us accepted that once we shared new knowledge through presentations and publications, the fate of that knowledge was mostly out of our hands. We understood, intuitively, that some new practices, such as laparoscopic cholecystectomy, might be adopted swiftly into surgical practices, driven by patient demand, while other practices, such as laparoscopic colectomy, might take decades to adopt, detained by a steep technical learning curve. We did not consider the possibility that we could, or should, systematically impact the speed of knowledge translation until the field of implementation science provided irrefutable evidence that there are long delays from the time research is published to the time it reaches practice; an average of 15 to 17 years for research and a range of 4 to 12 years for guidelines.^4^ Implementation science, fortunately, now provides a useful framework for promoting the clinical uptake of research. While it might be expected that deimplementation or discontinuation of overtreatment or low-value practices, based on evidence, might happen faster based on the concept of “do no harm,” that assumption has not been validated.

A contemporary experience from Wang et al^5^ illustrates the challenges associated with the real-world application of deimplementation strategies, in this case to achieve the goal of reducing rates of sentinel lymph node biopsy (SLNB) in women over the age of 70 and with hormone receptor positive cancer. Despite deliberate attention paid to critical steps in provider education, feedback on facility and provider-specific SLNB rates, and modeling through surgeon champions and reminders, the multifaceted, approach to deimplementation was associated with only modest impact despite 4 years of active interventions; SLNB rates fell from 78% in 2014 to 46% in 2018. Clearly if we are going to reduce waste and healthcare costs, we need to scale up, our strategies for deimplementation. Berlin et al^6^ assert that we can overcome barriers to deimplementation if we address the motivations that cause us to keep practicing low-value surgery, which may vary from financial incentives to misperceptions about risks and value and may originate from patients, professionals, and systems.^6^

Finally, one way of scaling up is to identify overtreatment and low-value practices that can be targeted for deimplementation, and that is where the Choosing Wisely campaign has taken the lead. This campaign, launched in 2012, now recommends against routine use of over 600 low-value healthcare services.^7^ The campaign focuses on improving quality and patient safety, with a core principle being the “just distribution of finite healthcare resources.” To avoid the risk of cost-cutting or healthcare rationing motivations, the campaign described a set of shared principles including that deimplementation efforts should be physician-led, patient-focused, evidence-based, multiprofessional, and transparent. While there may be many roads to reducing waste, including Choosing Wisely, it is clear that now is the time to start identifying low-value and overtreatment surgical practices and to start applying implementation science to the problem of eliminating these practices, to do no harm and to reduce the burden of cost.
